# A Brazilian Minamata disease? Neurologists must be aware of mercury exposure and intoxication

**DOI:** 10.1055/s-0045-1811622

**Published:** 2025-09-08

**Authors:** Gustavo Maximiano-Alves, Eder Leandro da Silva Dantas, Maria Elena Crespo-Lopez, José Luiz Martins do Nascimento

**Affiliations:** 1 Universidade de São Paulo, Faculdade de Medicina de Ribeirão Preto, Departamento de Neurociências e Ciências do Comportamento, Ribeirão Preto SP, Brazil.; 2 Universidade Federal do Pará, Instituto de Ciências Biológicas, Belém PR, Brazil.; 3 Instituto Amazônico do Mercúrio, Belém PA, Brazil.; 4 Instituto Nacional de Ciências e Tecnologia em Neuroimunologia, Rio de Janeiro RJ, Brazil.

**Keywords:** Mercury, Mercury Poisoning, Nervous System

## Abstract

Mercury intoxication poses a significant challenge and growing threat to public health, particularly in the Amazon region. Despite a known history of neurological damage, as evidenced by Japan's Minamata disease, mercury intoxication remains underdiagnosed in Brazil. This review underscores the need for increased clinical awareness among neurologists, as mercury exposure has been linked to over 250 neurological symptoms, including cognitive impairment, cerebellar ataxia, peripheral neuropathy, and psychiatric disturbances. The Indigenous and riverside populations in the Amazon present a high prevalence of cognitive and motor deficits, tremors, and sensory disturbances, which are associated with mercury body burdens. Diagnosis relies on a combination of clinical suspicion, environmental exposure history, and biomonitoring through hair and urine analyses. Given the widespread environmental contamination and potential long-term health consequences, neurologists must be vigilant in recognizing and managing mercury-related neurotoxicity, particularly in vulnerable Brazilian populations.

## THE USE OF MERCURY IN BRAZIL: AN ENVIRONMENTAL AND PUBLIC HEALTH TRAGEDY

The Amazon rainforest encompasses the world's largest river system, enormous biodiversity, and mineral resources that have been exploited for centuries. 1 Gold mining has been uncontrolled, increasing by almost 495% in Indigenous lands in the last decade. 2 3 Mining sites, known as *garimpo,* largely and illegally use mercury for gold extraction. 4 Currently, this is a large-scale activity that frequently uses heavy machinery, rather than “artisanal” or “small-scale” as described in international reports. 5 6 More than directly linked to deforestation, this process disrupts fishing, poisons the water, and can harm human health. 4 Consequently, mercury concentrations exceed permissible limits across multiple matrices, ranging from commercial fish to the hair of riverside populations. 7 8

Beyond these issues, there are also other ongoing public health concerns. The World Health Organization (WHO) has been supporting the phase-down use of mercury in dental care for more than 10 years. 9 Despite this, the use of encapsulated mercury-containing amalgams continues in Brazil. 10 Dental amalgams, even the encapsulated, have the potential of increasing the mercury concentration in blood, brain, and urine, which is linked to a high risk of infertility, inflammatory diseases, neurodevelopmental disorders, as well as Alzheimer's and Parkinson's diseases. 11 12 13 14 15 16 17 Inorganic mercury is also frequently detected in noncontrolled imported cosmetics, 18 19 which is a great problem, as Brazil is one of the world's largest beauty product consumers.

Responsible for a myriad of health issues, mercury intoxication has already been linked to different neurological symptoms. Considering the ongoing environmental tragedy, the use of mercury-containing amalgams and cosmetics, and the underdiagnosis of mercury intoxication in Brazil, 5 20 this review aims to alert neurologists to its clinical suspicion and differential diagnosis.

## NEUROLOGICAL ASPECTS

The Japanese Minamata basin tragedy was the first great awareness of methylmercury (MeHg) poisoning. Children exposed in utero and diagnosed with congenital Minamata disease had intellectual disabilities, cerebellar ataxia, failure to thrive, limb deformities, epilepsy, strabismus, and an increased prevalence of cerebral palsy. 21 22 Later, homemade bread prepared with grain treated with MeHg as a fungicide caused a severe outbreak of cases in Iraq. 21 In the face of these enormous public health issues, researchers and politicians started to debate the acute and chronic aspects of mercury neurotoxicity.

**Figure 1:**
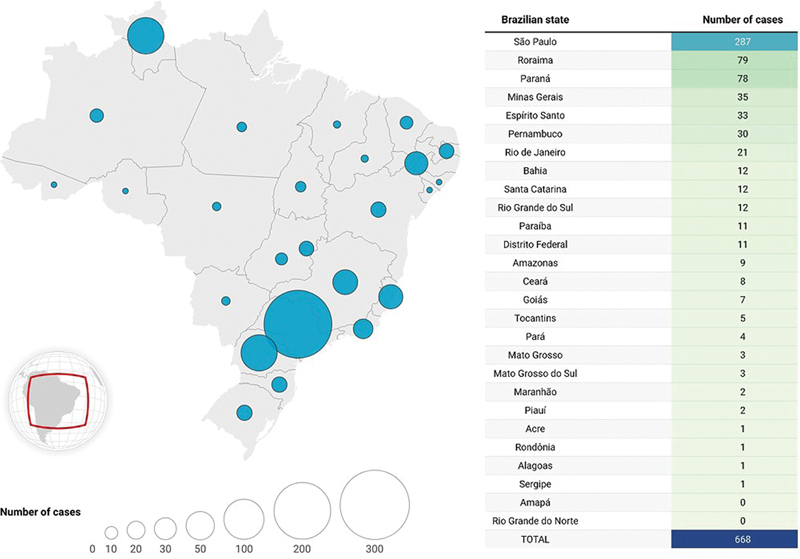
Distribution of reported cases in Brazil from 2007 to 2022. Source: Modified from Crespo-Lopez et al. 20 . The figure was elaborated using Datawrapper.

Although less widespread and resolutive than necessary, this discussion currently exists in Brazil. In 15 years, the Latin America's largest country registered more than 600 cases of mercury intoxication, 20 23 and most specialists assert the real number is even higher, due to underdiagnosis and notification problems. 5 20 As shown in Figure 1 , only 15.6% of officially notified cases occurred in the Amazon region, despite it harboring 92% of the country's gold mining activities. 20 This discrepancy suggests an alarming under-reporting of mercury intoxication in this region. Indeed, notwithstanding hundreds of cases in other Brazilian regions, there are no consistent published papers regarding intoxication's neurological symptoms or assessment.

**Figure 2:**
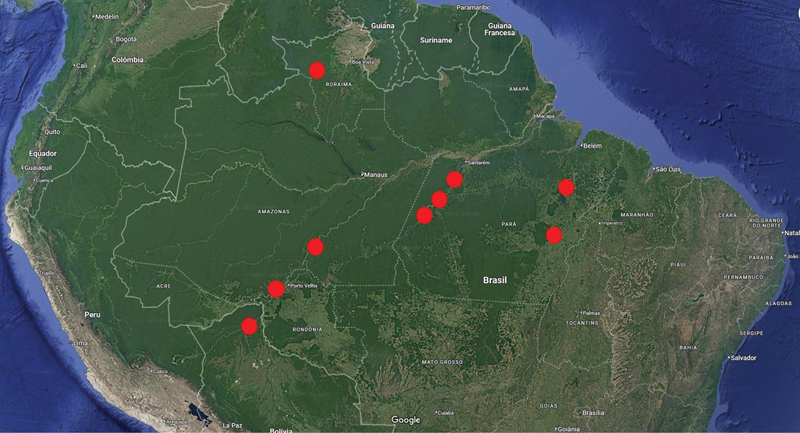
Map of the Amazon region. Geographical distribution of the clinical assessments reported in this review.

Despite its pulmonary, urinary, dermatological, and gastrointestinal effects, 24 this review will attain the neurological aspects of mercury intoxication, focusing on the national perspectives, particularly in the Amazon population, due to the underreported issues mentioned above ( Figure 2 ).

### From bench to clinics

Mercury can be found in three main species: the highly volatile elemental mercury (Hg ^0^ ), inorganic mercury (as found in mercury salts used in some cosmetics), and organic mercury (mainly MeHg). Elemental mercury has numerous uses in industry, such as fluorescent lamps, dental amalgams, and gold recovery, among others. The Hg ^0^ vapor, released into the air by these anthropogenic sources, is scattered by precipitation to soil and water (Hg ^2+^ ). 5 A bacterial biomagnification process transforms the inorganic mercury into MeHg, introducing it into the food chain. 5 Therefore, mercury reaches humans mainly by air (Hg ^0^ vapor) and food (MeHg intake), as well as by direct dermal contact (inorganic mercury and Hg ^0^ ), to a lesser degree. The main food contaminated with MeHg is seafood. The elemental species are mainly absorbed by the lungs, but also through the skin to a lesser extent. 21

Around 95% of the ingested MeHg is absorbed by the human body. 25 Once in the bloodstream, it is carried by hemoglobin and Hg ^0^ by cysteine/albumin/glutathione to the organs 25 and actively transported to the brain by the blood–brain barrier. 26 Furthermore, MeHg is slowly excreted, mainly by bile and feces, despite being detected in small amounts in human milk and urine. 21 It can also rapidly cross the placenta and transfer to the fetus. 21 All species of mercury are neurotoxic due to acute and/or chronic exposure. 21 However, this review will focus on the main ones (MeHg and Hg ^0^ ) responsible for the neurological outcomes of human intoxication. 27

The exposure pathway is relevant in addition to quantity. For example, inhaled Hg ^0^ rapidly crosses the blood–brain barrier when compared with the other mercury species and can lead to severe acute intoxication. 21 However, lower MeHg concentrations lead to worse outcomes, making it more neurotoxic than Hg ^0^ .

It is important to highlight that humans can be exposed to more than one mercury species simultaneously, through different pathways. For example, a *garimpo* worker can be exposed by inhalation during work, eating contaminated fish, and having their teeth repaired with mercury-containing amalgam. Gender, nutritional aspects, and other intraindividual factors, such as genetic polymorphisms and the microsomal system, could explain different physiopathological processes in the same population and interfere with the poisoning response. 21 28 29

**Figure 3:**
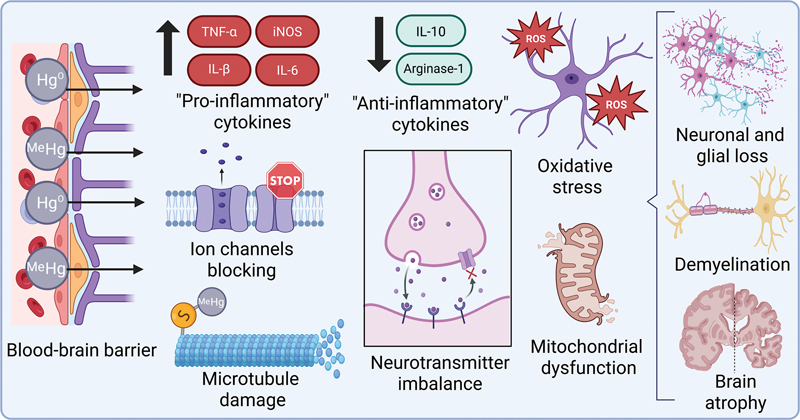
Illustration of the main molecular mechanisms of mercury neurotoxicity.

Mercury's neurotoxicity arises through multiple mechanisms, including the induction of oxidative stress, impairment of mitochondrial function, and disruption of neurotransmitter homeostasis. 30 In the peripheral nervous system, MeHg binds to the myelin sheath, damaging preferentially sensory neurons and dorsal root ganglia. 31 Additionally, there is documented evidence of degeneration in the granule and Purkinje cell layers of the cerebellum, 32 cerebral cortex, 33 and hippocampus ( Figure 3 ). 34

### Central nervous system

Exposure to MeHg in adults provokes focal central nervous system (CNS) impairment, while the developing brain is more susceptible to severe and global injury. 21 Congenital Minamata disease due to alimentary MeHg exposure is the most well-known neurological damage in children. All the affected newborns had intellectual disability, cerebellar ataxia, difficulty thriving, and limb deformities. 22 Most of them had epilepsy (82%), strabismus (77%), and pyramidal signs (75%), rather than the almost five times increased cerebral palsy prevalence. 21 22 Regarding national perspectives, studies are urgently needed to assess the real prevalence of congenital diseases among Brazilian children.

Cognitive impairment has also been proven in adults. Even basal mercury levels (1–2 ppm of hair mercury) due to chronic MeHg exposure have been significantly associated with worse results on rapid-processing cognitive tasks. 35 This diminished cognitive performance was correlated with a lower gray matter volume in the thalamus and hippocampus, as well as widespread reduced white matter volume, especially in the right basal ganglia and both frontal lobes. 35

Executive function involvement has already been confirmed in the Amazonian vulnerable populations. 27 In the Yanomami indigenous population living in the Northwest Amazon, there was a 95.9% higher prevalence of reduced cognitive performance among individuals with MeHg levels > 6.0 mcg/g in comparison to those with lower levels. 36 Also, Munduruku individuals of the Tapajós river basin with over 10 ppm of hair mercury have presented a 2-fold higher risk of alterations in the brief cognitive screening battery (BCSB) and verbal fluency (VF) test than those with lower MeHg levels. 37 Additionally, other vulnerable Amazonian populations, such as riverside communities, have registered cognitive alterations. In the Madeira river basin, children and adolescents with high mercury levels had lower scores in estimated intelligence quotient (IQ), visuospatial working memory, semantic knowledge, and phonological verbal fluency. 38

Behavioral abnormalities are other classic descriptions of acute or chronically intoxicated patients. Erethism (historically known as Mad Hatter syndrome) is the name given to increased excitability, emotional lability, and irritability related to mercury poisoning (especially Hg ^0^ ). 39 Other frequent neurological symptoms in acute Hg ^0^ poisoning are headache, nausea, abdominal pain, and distal limb paresthesia, as described in 179 pediatric Turkish cases. 40

Acute and chronic mercury exposure is also associated with several types of movement disorders. Cerebellar ataxia and dysarthria with cerebellar features are the most described findings. 41 Furthermore, tremors, cortical myoclonus, chorea, and Parkinsonism were reported worldwide. 42 In the Brazilian Amazon, a study involving gold traders detected a high frequency (6,6–10 Hz) of appendicular tremor. 43 In another interesting national investigation, chronically exposed gold miners were detected with higher rates of appendicular ataxia, nystagmus, and tremors. 44

### Visual system

Ophthalmological issues are pivotal in Minamata disease. Bilateral visual field constriction associated with visual acuity impairment was largely related to MeHg intoxication, 41 encompassing the classical description of the disorder. A possible explanation is the atrophy of calcarine fissures (primary visual cortex), which spares occipital poles, preserving the central vision. 45 46 47

In the Amazon population, impairment in color discrimination has been described as the main ophthalmologic finding, being associated with chronic MeHg contamination in adults 48 and children. 49 Additionally, there is a positive correlation between MeHg levels and the magnitude of the visual impairment, 49 diminished contrast sensitivity, 50 and reduced peripheral visual fields. 51

### Peripheral nervous system

Mercury serum levels are higher in patients diagnosed with idiopathic axonal neuropathy. However, the required damaging concentration and duration of exposure are still unknown. 52 Although electrophysiologic studies revealed consistent axonal polyneuropathy involving both motor and sensory fibers in a patient exposed to chronic inorganic mercury, 53 sensory disturbance (numbness and paresthesia), especially in Minamata's patients, is more attributable to sensory cortex damage than to peripheral nerve lesions. 54

Damaged afferent proprioceptive pathways could explain the higher prevalence of postural instability 55 and gait deviation with closed eyes in MeHg-contaminated individuals. 56 There is also a higher impairment in tactile sensation, two-point discrimination, and vibration in almost all body parts. 57 However, more neurophysiological assessments are necessary to analyze and discriminate large- and small-fiber involvement.

For instance, in the Yanonami population, it is estimated that approximately 30.3% of those with high levels of MeHg have some degree of peripheral neuropathy, mainly those with mercury levels above 6 µg/g. 36 In another Brazilian cohort, hand grip, manual dexterity, and muscular fatigue were also correlated with high MeHg levels. 58 These findings underscore the need for comprehensive national evaluations of mercury-associated peripheral neuropathy, employing a methodologically rigorous approach. Accurate diagnosis requires the exclusion of differential etiologies through the application of standardized diagnostic criteria for peripheral neuropathy, complemented by neurophysiological assessments. Such evaluations are essential to underly pathophysiological mechanisms and characterize patterns of nerve injury.

Despite rarity, subacute mercury poisoning due to skin-lightening creams and products from traditional Chinese medicine has already been related to complex neuromuscular findings. 59 A case series described patients presenting subacute fasciculation, cramps, myokymia, and neuromyotonia as a differential diagnosis of hyperexcitability disorders (Isaacs and Morvan syndromes). Motor neuron disease in an isolated form 60 61 was already described as associated with presynaptic myasthenic involvement, mimicking an overlap between amyotrophic lateral sclerosis and Lambert-Eaton syndrome. 59 Additionally, there is a report of vacuolar myopathy related to dental amalgams, 62 however, further descriptions in the literature concerning myopathy secondary to mercury intoxication are still scarce.

### Cardiovascular risk

According to the most robust evidence (systematic review and meta-analyses), more than 2 ppm of mercury in hair is associated with a 59% increase in the relative risk of hypertension and a significant increase in the risk of fatal and nonfatal outcomes related to cardiovascular diseases. 63 This is alarming considering that current chronic exposure to MeHg in Brazil can be 15 to 100 times higher (∼30 μg/L of blood mercury) 64 65 than reported in some international cohorts. 66 67 68

In specific Brazilian Munduruku Indigenous villages, a positive correlation between mercury intoxication and high blood pressure in pregnant women was reported. 69 Cross-sectional studies 64 found dyslipidemia and high/moderate cardiovascular risk correlated with hair mercury levels in chronically exposed riverside populations in the Amazon. Furthermore, a high prevalence of hypertension, metabolic syndrome, and a high risk of acute myocardial infarction have been registered in exposed populations of different Amazonian basins. 70 71 Neurologists need to be alerted to the early screening of these patients due to the increased risk of stroke.

### Miscellaneous findings

Bilateral hearing impairment was described and, despite cochlear involvement, some pathological studies found that the primary auditory area of the temporal lobe was affected, which is probably the main etiology. 54 A perioral sensory disturbance, described as an “onion peel,” is common, indicating somatosensory cortical involvement instead of peripheral damage. 41

Other documented symptoms in exposed individuals living in the Amazon were insomnia, depression, anxiety, limb pain, 72 and partial hearing loss. 44 However, to this date, these findings lack a statistically significant correlation with mercury levels and differential diagnosis.

### Neuroimaging

Brain magnetic resonance imaging (MRI) may show gray matter volume reduction in cerebellum, calcarine fissure, and thalamus. 73 Exposure time is associated with different patterns, with the thalamic atrophy being most evident in fetuses (contaminated since the intrauterine life), while cerebellar and calcarine fissure abnormalities are more commonly observed in those affected during childhood or adult life. 73 Some case reports showed reversible, subcortical, white matter T2-hyperintense lesions in acutely intoxicated children. 74 75 Nonetheless, the main neuroimaging application in mercury intoxication is helping to rule out the differential diagnoses.

## WHEN TO SUSPECT

Neurologists should always raise suspicion when consulting a patient presenting the constellation of symptoms mentioned above ( Table 1 ) reporting previous exposure to a potential source of mercury (such as high intake of fish, presence of dental amalgams, use of imported cosmetics, work in *garimpos* , use of thermometers and batteries) given special attention to the Amazon populations. 7 8 76 Another interesting point is that neurological symptoms could appear at least 20 years after exposure, as described in the classical Minamata disease 21 and the anamnesis is hugely important at this moment to establish causal correlation. Child neurologists should screen for mercury intoxication in patients presenting CNS lesions (cerebral-palsy-like) without the most prevalent acquired and genetic causes. A careful investigation into patients' history is needed, questioning the mothers about environmental/occupational exposure.

**Table 1:** Main neurological findings in chronic mercury intoxication described in this review

	Signs and symptoms	References
Cognition and behavioral	Reduced performance in BCSB and the verbal fluency test, and erethism (increased excitability, emotional lability, and irritability).	37 39
Ophthalmological	Bilateral visual constriction, color discrimination deficit, and reduced contrast sensitivity.	41 48 50
Neuromuscular	Sensory axonal neuropathy with preserved deep tendon reflexes, numbness, and paresthesia.	36 41 57
Movement disorders	High-frequency tremor (6.6–10 Hz), cerebellar ataxia. Others (see text, less frequent).	41 43 44
Miscellanea	Bilateral hearing impairment, “onion peel” perioral paresthesia.	41 44 54
Neurodevelopmental and congenital disease	Intellectual disability, failure to thrive, limb deformities, epilepsy, strabismus, pyramidal signs, cerebral palsy-like.	22

Abbreviations: BCSB, brief cognitive screening battery; Hz, Hertz.

No case of mercury poisoning from drinking contaminated water has ever been described because it is hardly found in sufficient quantities, and direct gastrointestinal absorption of inorganic mercury (as mainly found in water) is low. However, any presence of mercury in water influences the contamination of the food chain. 7 76 Higher levels of mercury are currently found in the riverside population living near the Tucuruí hydroelectric dam (in the Tocantins river region, which is not influenced by gold mining). 29 64 71

It is worth noting that the consequences of mining can extend far beyond the extraction site, and clinical suspicion should not be restricted to people living closer to mining areas or in the Amazon region. Any Brazilian inhabitant who frequently consumes fish and seafood may be exposed to organic mercury. Almost all Brazilian states have already registered confirmed or suspected cases of acute or chronic exposure/intoxication ( Figure 1 ). 5 20 Thus, this should concern all Brazilian health professionals who work with neurological diseases.

## DIAGNOSIS

There is no pathognomonic test. The suspected cases must be analyzed from the cluster of symptoms ( Table 1 ), 21 previous personal exposure, analyses of mercury body burden, and correlation analysis of risk factors and differential diagnosis. The 1977 Diagnostic Criteria of Minamata Disease may be used, but its accuracy is debated 77 and there is no validation data in the Brazilian population. After the initial suspicion, the physician must fill in a compulsory notification form alerting the regional health secretary. Then, local authorities are officially obligated to investigate epidemiological risks and perform laboratory analyses. 78

Urinary mercury is indicative of exposure mainly to Hg ^0^ , as a significant part is transformed into inorganic mercury and eliminated in the urine. Otherwise, hair mercury indicates exposure primarily to MeHg, as approximately 10% of this species is deposited in the hair, which is directly correlated with the brain load.

Unlike blood analysis, both urinary and hair analyses are noninvasive and informative methods. Blood mercury is from any pathway of exposure, being therefore nonspecific). As such, mercury analysis in hair is less expensive and has good correlations, as MeHg is slowly accumulated over time. 79 The hair from the initial 0.5 cm next to the scalp represents, on average, MeHg exposure during the 3 weeks before the collection date ( **Supplementary Material** – available at https://www.arquivosdeneuropsiquiatria.org/wp-content/uploads/2025/06/ANP-2025.0161-Supplementary-Material.docx ).

For the diagnosis and interpretation of laboratory results, it is essential to understand that, in chronic mercury exposure, the levels fluctuate over time, increasing when a new load received and subsequently decreasing through metabolism and the metal's clearance. 79 Therefore, considering the coexposure risk, patients should be assessed using both urinary and hair analyses. Those with negative levels and high clinical suspicion must be followed by the doctor and have their laboratory reassessed later. Another essential point is that mercury levels do not necessarily correlate with symptoms, considering the latency since the start of exposure (sometimes of decades, as described before). Therefore, patients presenting high levels of mercury without evident signs or symptoms must also be monitored.

The WHO recommends a provisional tolerable weekly intake (PTWI) for MeHg of 1.6 µg/Kg body weight (see **Supplementary References** ), which is approximately equivalent to 2.3 mg/Kg of hair mercury. 7 Other agencies such as the Environmental Protection Agency of the United States (USEPA) and the National Institute for Public Health and the Environment of the Netherlands (NIPHE) are more cautious, and they recommend a PTWI of 0.7 µg/Kg body weight (equivalent to 1.0 mg/Kg of hair mercury). 8 Despite these recommendations, it is crucial to be aware of the individual factors (genetic, nutritional, occupational, coexposure, etc.) influencing the neurological outcomes. Therefore, any mercury concentration has the potential to be neurotoxic, requiring a case-by-case analysis (see **Supplementary References** ).

## TREATMENT

In acute poisoning, patients require inpatient evaluation and emergency care. Gastric lavage and activated charcoal are possible prescriptions, but their value has been challenged and lacks clinical evidence (see **Supplementary References** ). Hemodialysis could be a helpful strategy for severely ill patients (see **Supplementary References** ). Special attention to pulmonary complications must be offered for those with acute Hg ^0^ vapor exposure by inhalation (see **Supplementary References** ).

To date, no controlled clinical study has demonstrated the efficacy of the treatment with chelation agents in mercury intoxication (see **Supplementary References** ). Due to the high adverse effects of the use of these agents, the risk-benefit therapeutic index must be carefully evaluated in each case. Chelating agents with thiol groups (dimercaptosuccinic acid, DMSA, and N-acetyl-D-L-penicillamine) have already been used for acute intoxication, but their real effect has been questioned, and more studies are needed (see **Supplementary References** ). Furthermore, chelation therapy can only decrease the mercury level in blood, without any direct impact on symptoms already established, especially neurological outcomes. The availability of these agents in the Brazilian health system are another challenge.

For chronic intoxication, it must be highlighted that chelation therapy is not recommended, as there is no clinical evidence of efficacy. Furthermore, the presence of side effects was noted. Indeed, the main therapy remains to remove the patient from the source of exposure and provide them with health support. Chronic patients with cognitive, psychiatric, and neurological sequelae can be treated with psychotropic and relief medications. Unfortunately, there is no therapy to reduce neuronal damage after long exposure. 41

## IS THERE A BRAZILIAN MINAMATA DISEASE?

Especially on Amazon, numerous epidemiological studies 27 have suggested neurological outcomes associated with mercury exposure and intoxication. Considering the spread of this pollutant, a national assessment is urgent, with Hg ^0^ remaining airborne for a long time and travel long distances, and fish with MeHg being sold in national and international markets. There is an enormous lack of information owing to underdiagnosis and unawareness among the population and doctors. The number of exposed people is higher than official records, as demonstrated in the Amazon by comparing Brazilian government data 5 with that of scientific literature. 27 Unfortunately, there are newborns and elderly people struggling with neurological conditions without knowing the name of their disease.

Adult and child neurologists must raise their suspicions and contribute to case notification. More patients must be tested for mercury exposure inside and outside the Amazon river basin. At the same time, the population should be enlightened about the symptoms and the disease progression. All patients presenting with acute or chronic suggestive features ( Table 1 ) and potential exposure (Amazonian populations, consumption of contaminated fish and seafood, dental amalgams, and illegal cosmetics) with no other common/clear etiology for the symptoms must be notified and tested for mercury exposure.

Despite all the alerts from toxicologists, politicians continue to turn a blind eye to this issue. As health professionals, we cannot do the same. Initiatives must be supported, such as the bill proposal n° 1011/2023 for the implementation of National Policies to Prevent Mercury Exposure in Brazil, engaging collaborative efforts of Amazonian public institutions, especially universities, to increase mercury monitoring and prevention strategies. It is important to engage in collaborative efforts and increase mercury monitoring, enhance diagnosis, and raise prevention strategies. Then, the course of this announced tragedy might change.

Additional references are available in the “ **Supplementary References** ” section provided online.

## References

[1] Putzer H The geological evolution of the Amazon basin and its mineral resources Dordrecht Springer Nature 1984 15 4610.1007/978-94-009-6542-3_2

[2] MapBiomas Brasil . Área ocupada pela mineração no Brasil cresce mais de 6 vezes entre 1985 e 2020. Accessed March 11, 2025.https://brasil.mapbiomas.org/2021/08/30/area-ocupada-pela-mineracao-no-brasil-cresce-mais-de-6-vezes-entre-1985-e-2020/

[3] Ellwanger J H Chies J AB Brazil's heavy metal pollution harms humans and ecosystems Sci One Health 2023 2 100019 10.1016/j.soh.2023.100019 39077034 PMC11262263

[4] Ferreira Neto L C Diniz C G Maretto R V Persello C Pinheiro M LS Castro M C Uncontrolled Illegal Mining and Garimpo in the Brazilian Amazon Nat Commun 2024 15 01 9847 10.1038/s41467-024-54220-2 39537611 PMC11560919

[5] Crespo-Lopez M E Arrifano GdP Augusto-Oliveira M Araújo A L Sacramento LdS Barthelemy J L Souza CBAd Mineração e Mercúrio na Amazônia: principais perguntas e respostas(orgs.). Mineração de Ouro Artesanal e em Pequena Escala na Amazônia - Grandes Impactos Socioambientais e Violações Multidimensionais de Direitos Humanos. Manaus: UEA,2023 15 70

[6] Crespo-Lopez M E Arrifano G P Augusto-Oliveira M Macchi B M Lima R R do Nascimento J LM de Souza C BA Mercury in the Amazon: The danger of a single story Ecotoxicol Environ Saf 2023 256 114895 10.1016/J.ECOENV.2023.114895 37062263

[7] Martín-Doimeadios R CR Nevado J JB Bernardo F JG Moreno M J Arrifano G PF Herculano A M Comparative study of mercury speciation in commercial fishes of the Brazilian Amazon Environ Sci Pollut Res Int 2014 21 12 7466 7479 10.1007/S11356-014-2680-7 24590602

[8] Crespo-Lopez M E Augusto-Oliveira M Lopes-Araújo A Santos-Sacramento L Takeda P Y Macchi BdM Mercury: What can we learn from the Amazon? Environ Int 2021 146 106223 10.1016/j.envint.2020.106223 33120229

[9] World Health Organization . Future use of materials for dental restoration: Report of the meeting at WHO Headquarters, Geneva, Switzerland, 16–17 November 2009. Geneva: World Health Organization,2011. 65 p

[10] ANVISA . Agência Nacional de Vigilância Sanitária. Resolução da Diretoria Colegiada – RDC No 879, de 28 de Maio de2024. Brazil (2024)

[11] World Health Organization . Mercury and health. World Health Organization (WHO)

[12] Geier D A Geier M R Dental Amalgams and the Incidence Rate of Arthritis among American Adults Clin Med Insights Arthritis Musculoskelet Disord 2021 14 11795441211016261. 10.1177/.11795441211016261 PMC8138300 34045912

[13] Geier D A Geier M R Reported asthma and dental amalgam exposure among adults in the United States: An assessment of the National Health and Nutrition Examination Survey SAGE Open Med 2021 9 20503121211048677. 10.1177/20503121211048677 PMC8532208 34691469

[14] Geier D A Geier M R Dental amalgam fillings and mercury vapor safety limits in American adults Hum Exp Toxicol 2022 41 9.60327122110634E1510.1177/09603271221106341 10.1177/09603271221106341 35786065

[15] Zhu F Chen C Zhang Y Chen S Huang X Li J Elevated blood mercury level has a non-linear association with infertility in U.S. women: Data from the NHANES 2013-2016 Reprod Toxicol 2020 91 53 58 10.1016/j.reprotox.2019.11.005 31756438

[16] Tibau A Grube B Alarming Findings on Mercury Dental Amalgam – Latest Research Using the National Health and Nutrition Examination Survey (NHANES) Database: A Mini-Review Oral Health Dental Sci 2022 6 02 1 6

[17] Mutter J Is dental amalgam safe for humans? The opinion of the scientific committee of the European Commission J Occup Med Toxicol 2011 6 01 2 10.1186/1745-6673-6-2 21232090 PMC3025977

[18] Zero Mercury Wotking Group (ZMWG) . Skin Lighteners Still Online Despite Mercury Findings. 2022.https://www.zeromercury.org/skin-lighteners-still-online-despite-mercury-findings/

[19] Crespo-Lopez M E Augusto-Oliveira M Lopes-Araújo A Santos-Sacramento L Souza-Monteiro J R da Rocha F F Arrifano GdP Mercury neurotoxicity in gold miners Adv Neurotoxicol 2022 7 283 314 10.1016/bs.ant.2022.04.003

[20] Crespo-Lopez M E Lopes-Araújo A Basta P C Soares-Silva I de Souza C BA Leal-Nazaré C G Environmental pollution challenges public health surveillance: the case of mercury exposure and intoxication in Brazil Lancet Reg Health Am 2024 39 100880 10.1016/j.lana.2024.100880 39290578 PMC11402532

[21] National Research Council (US) Committee on the Toxicological Effects of Methylmercury . Chemistry, exposure, toxicokinetics, and toxicodynamics. In: Toxicological Effects of Methylmercury. Washington: National Academies Press; 2000. Accessed March 11, 2025.https://www.ncbi.nlm.nih.gov/books/NBK225779

[22] Harada M Minamata disease: methylmercury poisoning in Japan caused by environmental pollution Crit Rev Toxicol 1995 25 01 1 24 10.3109/10408449509089885 7734058

[23] Ministério da Saúde . Boletim Epidemiológico. Secretaria de Vigilância em Saúde e Ambiente, Brasil. 2023;54(02)

[24] Kim K H Kabir E Jahan S A A review on the distribution of Hg in the environment and its human health impacts J Hazard Mater 2016 306 376 385 10.1016/j.jhazmat.2015.11.031 26826963

[25] Bradley M A Barst B D Basu N A Review of Mercury Bioavailability in Humans and Fish Int J Environ Res Public Health 2017 14 02 169 10.3390/ijerph14020169 28208586 PMC5334723

[26] Kerper L E Ballatori N Clarkson T W Methylmercury transport across the blood-brain barrier by an amino acid carrier Am J Physiol 1992 262(5 Pt 2):R761 R765 10.1152/ajpregu.1992.262.5.R761 1590471

[27] Santos-Sacramento L Arrifano G P Lopes-Araújo A Augusto-Oliveira M Albuquerque-Santos R Takeda P Y Human neurotoxicity of mercury in the Amazon: A scoping review with insights and critical considerations Ecotoxicol Environ Saf 2021 208 111686 10.1016/j.ecoenv.2020.111686 33396018

[28] Arrifano G P Augusto-Oliveira M Sealey-Bright M Zainal J Imbiriba L Fernandes L MP Contributing to Understand the Crosstalk between Brain and Periphery in Methylmercury Intoxication: Neurotoxicity and Extracellular Vesicles Int J Mol Sci 2021 22 19 10855 10.3390/ijms221910855 34639196 PMC8509412

[29] Arrifano G PF Martín-Doimeadios R CR Jiménez-Moreno M Fernández-Trujillo S Augusto-Oliveira M Souza-Monteiro J R Genetic susceptibility to neurodegeneration in Amazon: Apolipoprotein E genotyping in vulnerable populations exposed to mercury Front Genet 2018 9 285 10.3389/fgene.2018.00285 30100920 PMC6073741

[30] Kang B Wang J Guo S Yang L Mercury-induced toxicity: Mechanisms, molecular pathways, and gene regulation Sci Total Environ 2024 943 173577 10.1016/j.scitotenv.2024.173577 38852866

[31] Nakano T Yoshida E Sasaki Y Kazama S Katami F Aoki K Mechanisms Underlying Sensory Nerve-Predominant Damage by Methylmercury in the Peripheral Nervous System Int J Mol Sci 2024 25 21 11672 10.3390/ijms252111672 39519224 PMC11545846

[32] Bittencourt L O Chemelo V S Aragão W AB Puty B Dionizio A Teixeira F B From Molecules to Behavior in Long-Term Inorganic Mercury Intoxication: Unraveling Proteomic Features in Cerebellar Neurodegeneration of Rats Int J Mol Sci 2021 23 01 111 10.3390/ijms23010111 35008538 PMC8745249

[33] Teixeira F B de Oliveira A CA Leão L KR Fagundes N CF Fernandes R M Fernandes L MP Exposure to inorganic mercury causes oxidative stress, cell death, and functional deficits in the motor cortex Front Mol Neurosci 2018 11 12510.3389/fnmol.2018.00125 29867340 10.3389/fnmol.2018.00125 PMC5962769

[34] Teixeira F B Fernandes R M Farias-Junior P MA Costa N MM Fernandes L MP Santana L NS Evaluation of the effects of chronic intoxication with inorganic mercury on memory and motor control in rats Int J Environ Res Public Health 2014 11 09 9171 9185 10.3390/ijerph110909171 25198682 PMC4199013

[35] Takeuchi H Shiota Y Yaoi K Taki Y Nouchi R Yokoyama R Mercury levels in hair are associated with reduced neurobehavioral performance and altered brain structures in young adults Commun Biol 2022 5 01 529 10.1038/s42003-022-03464-z 35655003 PMC9163068

[36] Rebouças B H Kubota G T Oliveira R AA Pinto B D Cardoso R M Vasconcellos A CS Basta P C Long-Term Environmental Methylmercury Exposure Is Associated with Peripheral Neuropathy and Cognitive Impairment among an Amazon Indigenous Population Toxics 2024 12 03 212 10.3390/toxics12030212 38535945 PMC11154458

[37] Oliveira RAAd Pinto B D Rebouças B H de Andrade D C Vasconcellos ACSd Basta P C Neurological Impacts of Chronic Methylmercury Exposure in Munduruku Indigenous Adults: Somatosensory, Motor, and Cognitive Abnormalities Int J Environ Res Public Health 2021 18 19 10270 10.3390/ijerph181910270 34639574 PMC8507861

[38] Santos-Lima Cd Mourão DdS Carvalho CFd Souza-Marques B Vega C M Gonçalves R A Neuropsychological Effects of Mercury Exposure in Children and Adolescents of the Amazon Region, Brazil Neurotoxicology 2020 79 48 57 10.1016/j.neuro.2020.04.004 32335201

[39] Ozuah P O Mercury poisoning Curr Probl Pediatr 2000 30 03 91 99 10.1067/mps.2000.104054 10742922

[40] Carman K B Tutkun E Yilmaz H Dilber C Dalkiran T Cakir B Acute mercury poisoning among children in two provinces of Turkey Eur J Pediatr 2013 172 06 821 827 10.1007/s00431-013-1970-2 23411638

[41] Jackson A C Chronic Neurological Disease Due to Methylmercury Poisoning Can J Neurol Sci 2018 45 06 620 623 10.1017/cjn.2018.323 30278852

[42] Ganguly J Kulshreshtha D Jog M Mercury and Movement Disorders: The Toxic Legacy Continues Can J Neurol Sci 2022 49 04 493 501 10.1017/cjn.2021.146 34346303

[43] Biernat H Ellias S A Wermuth L Cleary D Santos ECdO Jørgensen P J Tremor frequency patterns in mercury vapor exposure, compared with early Parkinson's disease and essential tremor Neurotoxicology 1999 20 06 945 952 10693975

[44] Corbett C EP El Khouri M Costa A N Gyuricza J V Corbett J F Frizzarini R Health evaluation of gold miners living in a mercury-contaminated village in Serra Pelada, Pará, Brazil Arch Environ Occup Health 2007 62 03 121 128 10.3200/aeoh.62.3.121-128 18400651

[45] Hunter D Russell D S Focal cerebellar and cerebellar atrophy in a human subject due to organic mercury compounds J Neurol Neurosurg Psychiatry 1954 17 04 235 241 10.1136/jnnp.17.4.235 13212411 PMC503192

[46] Korogi Y Takahashi M Okajima T Eto K MR findings of Minamata disease--organic mercury poisoning J Magn Reson Imaging 1998 8 02 308 316 10.1002/jmri.1880080210 9562057

[47] Yorifuji T Tsuda T Takao S Harada M Long-term exposure to methylmercury and neurologic signs in Minamata and neighboring communities Epidemiology 2008 19 01 3 9 10.1097/EDE.0b013e31815c09d2 18091411

[48] Feitosa-Santana C Souza GdS Sirius E VP Rodrigues A R Cortes M IT Silveira LCdL Ventura D F Color vision impairment with low-level methylmercury exposure of an Amazonian population - Brazil Neurotoxicology 2018 66 179 184 10.1016/j.neuro.2018.01.010 29432854

[49] Freitas JdS Lacerda EMdCB Martins ICVdS Rodrigues Jr D Bonci D MO Cortes M IT Cross-sectional study to assess the association of color vision with mercury hair concentration in children from Brazilian Amazonian riverine communities Neurotoxicology 2018 65 60 67 10.1016/j.neuro.2018.02.006 29428869

[50] Rodrigues A R Souza C RB Braga A M Rodrigues P SS Silveira A T Damin E TB Mercury toxicity in the Amazon: contrast sensitivity and color discrimination of subjects exposed to mercury Braz J Med Biol Res 2007 40 03 415 424 10.1590/s0100-879x2007000300018 17334540

[51] Lebel J Mergler D Lucotte M Amorim M Dolbec J Miranda D Evidence of early nervous system dysfunction in Amazonian populations exposed to low-levels of methylmercury Neurotoxicology 1996 17 01 157 167 8784826

[52] Latov N Kumar G Vo M L Chin R L Carey B T Langsdorf J A Feuer N T Elevated blood mercury levels in idiopathic axonal neuropathy JAMA Neurol 2015 72 04 474 475 10.1001/jamaneurol.2015.1 25867721

[53] Chu C C Huang C C Ryu S J Wu T N Chronic inorganic mercury induced peripheral neuropathy Acta Neurol Scand 1998 98 06 461 465 10.1111/j.1600-0404.1998.tb07331.x 9875628

[54] Ekino S Susa M Ninomiya T Imamura K Kitamura T Minamata disease revisited: an update on the acute and chronic manifestations of methyl mercury poisoning J Neurol Sci 2007 262(1-2):131 144 10.1016/j.jns.2007.06.036 17681548

[55] Benefice E Luna-Monrroy S Lopez-Rodriguez R Fishing activity, health characteristics and mercury exposure of Amerindian women living alongside the Beni River (Amazonian Bolivia) Int J Hyg Environ Health 2010 213 06 458 464 10.1016/j.ijheh.2010.08.010 20851675

[56] Khoury E DT Souza GdS Silveira LCdL Costa C A Araújo A A Pinheiro MdCN Neurological manifestations in riverine populations from areas exposed to mercury in the Brazilian Amazon [Manifestações neurológicas em ribeirinhos de áreas expostas ao mercúrio na Amazônia brasileira] Cad Saude Publica 2013 29 11 2307 2318 10.1590/0102-311Doi:00158012 24233045

[57] Khoury E DT Souza GdS Costa CAd de Araújo A AK Oliveira CSBd Silveira LCdL Pinheiro MdCN Somatosensory Psychophysical Losses in Inhabitants of Riverside Communities of the Tapajós River Basin, Amazon, Brazil: Exposure to Methylmercury Is Possibly Involved PLoS One 2015 10 12 e0144625 10.1371/journal.pone.0144625 26658153 PMC4676688

[58] Lebel J Mergler D Branches F Lucotte M Amorim M Larribe F Dolbec J Neurotoxic effects of low-level methylmercury contamination in the Amazonian Basin Environ Res 1998 79 01 20 32 10.1006/enrs.1998.3846 9756677

[59] Zhou Z Zhang X Cui F Liu R Dong Z Wang X Yu S Subacute motor neuron hyperexcitability with mercury poisoning: a case series and literature review Eur Neurol 2014 72(3-4):218 222 10.1159/000363290 25227723

[60] Singh D Lal V Mercury masquerading as motor neuron disease J Neurol Sci 2021 429 119389 10.1016/j.jns.2021.119389

[61] Pamphlett R Jew S K Heavy metals in locus ceruleus and motor neurons in motor neuron disease Acta Neuropathol Commun 2013 12 1 81 10.1186/2051-5960-1-81 PMC3878779 24330485

[62] Akbal A Yılmaz H Tutkun E Köş D M Aggravated neuromuscular symptoms of mercury exposure from dental amalgam fillings J Trace Elem Med Biol 2014 28 01 32 34 10.1016/j.jtemb.2013.09.005 24210170

[63] Hu X F Lowe M Chan H M Mercury exposure, cardiovascular disease, and mortality: A systematic review and dose-response meta-analysis Environ Res 2021 193 110538 10.1016/j.envres.2020.110538 33285155

[64] Lopes-Araújo A Arrifano G P Macchi B M Augusto-Oliveira M Santos-Sacramento L Martín-Doimeadios R CR Hair mercury is associated with dyslipidemia and cardiovascular risk: An anthropometric, biochemical and genetic cross-sectional study of Amazonian vulnerable populations Environ Res 2023 229 115971 10.1016/j.envres.2023.115971 37105291

[65] Castro NSSd Lima MdO Hair as a Biomarker of Long Term Mercury Exposure in Brazilian Amazon: A Systematic Review Int J Environ Res Public Health 2018 15 03 500 10.3390/ijerph15030500 29534534 PMC5877045

[66] Sun Y Liu B Rong S Zhang H Du Y Xu G Association of Seafood Consumption and Mercury Exposure with Cardiovascular and All-Cause Mortality Among US Adults JAMA Netw Open 2021 4 11 e2136367 10.1001/jamanetworkopen.2021.36367 34842923 PMC8630568

[67] Chen C Xun P McClure L A Brockman J MacDonald L Cushman M Serum mercury concentration and the risk of ischemic stroke: The REasons for Geographic and Racial Differences in Stroke Trace Element Study Environ Int 2018 117 125 131 10.1016/j.envint.2018.05.001 29738916 PMC5997556

[68] Golding J Hibbeln J R Gregory S M Iles-Caven Y Emond A Taylor C M Maternal prenatal blood mercury is not adversely associated with offspring IQ at 8 years provided the mother eats fish: A British prebirth cohort study Int J Hyg Environ Health 2017 220 07 1161 1167 10.1016/j.ijheh.2017.07.004 28754500 PMC5584731

[69] Basta P C Viana P VS Vasconcellos A CS Périssé A RS Hofer C B Paiva N S Mercury Exposure in Munduruku Indigenous Communities from Brazilian Amazon: Methodological Background and an Overview of the Principal Results Int J Environ Res Public Health 2021 18 17 9222 10.3390/ijerph18179222 34501811 PMC8430525

[70] Machado C LR Crespo-Lopez M E Augusto-Oliveira M Arrifano GdP Macchi BdM Lopes-Araújo A Eating in the Amazon: Nutritional Status of the Riverine Populations and Possible Nudge Interventions Foods 2021 10 05 1015 10.3390/foods10051015 34066557 PMC8148567

[71] Arrifano G PF Alvarez-Leite J I Souza-Monteiro J R Augusto-Oliveira M Paraense R Macchi B M In the Heart of the Amazon: Noncommunicable Diseases and Apolipoprotein E4 Genotype in the Riverine Population Int J Environ Res Public Health 2018 15 09 1957 10.3390/ijerph15091957 30205523 PMC6165059

[72] Costa J MF Lima A ADS Rodrigues Junior D Khoury E DT Souza GdS Silveira LCdL Pinheiro MdCN Emotional and motor symptoms in riverside dwellers exposed to mercury in the Amazon [Manifestações emocionais e motoras de ribeirinhos expostos ao mercúrio na Amazônia] Rev Bras Epidemiol 2017 20 02 212 224 10.1590/1980-5497201700020003 28832845

[73] Hirai T Abe O Nakamura M Inui S Uetani H Ueda M Azuma M Brain structural changes in patients with chronic methylmercury poisoning in Minamata Brain Res 2023 15 1805 148278 10.1016/j.brainres.2023.148278 36775085

[74] Benz M R Lee S H Kellner L Döhlemann C Berweck S Hyperintense lesions in brain MRI after exposure to a mercuric chloride-containing skin whitening cream Eur J Pediatr 2011 170 06 747 750 10.1007/S00431-010-1333-1 21052738

[75] Abbaslou P Zaman T A Child with elemental mercury poisoning and unusual brain MRI findings Clin Toxicol (Phila) 2006 44 01 85 88 10.1080/15563650500394969 16496500

[76] Basta P C Vasconcellos ACSd Hallwass G Yokota D Pinto D ODR Aguiar DSd Risk Assessment of Mercury-Contaminated Fish Consumption in the Brazilian Amazon: An Ecological Study Toxics 2023 11 09 800 10.3390/toxics11090800 37755810 PMC10535031

[77] Yorifuji T Tsuda T Inoue S Takao S Harada M Kawachi I Critical appraisal of the 1977 diagnostic criteria for Minamata disease Arch Environ Occup Health 2013 68 01 22 29 10.1080/19338244.2011.627894 23298421

[78] Ministério da Saúde; Secretaria de Vigilância em Saúde; Departamento de Saúde Ambiental do Trabalhador e de Vigilância das Emergências em Saúde Pública . Orientações Para a Notificação de Intoxicações por Mercúrio. Brasília: Ministério da Saúde, 2021.https://www.gov.br/saude/pt-br/centrais-de-conteudo/publicacoes/svsa/vigilancia-ambiental/orientacoes-para-notificacao-intoxicacao-por-mercurio/view

[79] Al-Shahristani H Shihab K Al-Haddad I K Mercury in hair as an indicator of total body burden Bull World Health Organ 1976 53(Suppl):105 112 1086158 PMC2366395

[80] Nuttall K L Interpreting Hair Mercury Levels in Individual Patients Ann Clin Lab Sci 2006 36 03 248 261 16951265

[81] World Health Organization . Guidance for identifying populations at risk from mercury exposure. Geneva: UNEP DTIE Chemicals Branch and WHO Department of Food Safety, Zoonoses and Foodborne Diseases,2008. Available from:https://www.who.int/foodsafety/publications/chem/mercuryexposure.pdf. Last accessed March 2025

[82] Sue Y Mercury 10th ed. Colombus McGraw-Hill 2015

[83] Agocs M. Case studies in environmental medicine: Mercury toxicity. Agency for Toxic Substances and Disease Registry; US Department of Health & Human Services, 1992

[84] Ye B J Kim B G Jeon M J Kim S Y Kim H C Jang T W Evaluation of mercury exposure level, clinical diagnosis and treatment for mercury intoxication Ann Occup Environ Med 2016 28 01 5 10.1186/S40557-015-0086-8 26807265 PMC4724159

[85] Elinder C G Friberg L Kjellström T Nordberg G F Oberdoerster GWorld Health Organization. Biological monitoring of metals. Geneva: Chemical Safety Monographs International Program, 1994. 78 p

